# Computer-Aided Drug Design Applied to Marine Drug Discovery: Meridianins as Alzheimer’s Disease Therapeutic Agents

**DOI:** 10.3390/md15120366

**Published:** 2017-11-27

**Authors:** Laura Llorach-Pares, Alfons Nonell-Canals, Melchor Sanchez-Martinez, Conxita Avila

**Affiliations:** 1 Department of Evolutionary Biology, Ecology and Environmental Sciences, Faculty of Biology and Biodiversity Research Institute (IRBio), Universitat de Barcelona, 08028 Barcelona, Catalonia, Spain; laura@mindthebyte.com; 2 Mind the Byte S.L., 08028 Barcelona, Catalonia, Spain; alfons@mindthebyte.com

**Keywords:** computer-aided drug discovery/design, meridianins, Alzheimer disease, protein kinases, tau protein kinases, dual specificity kinases, marine natural products

## Abstract

Computer-aided drug discovery/design (CADD) techniques allow the identification of natural products that are capable of modulating protein functions in pathogenesis-related pathways, constituting one of the most promising lines followed in drug discovery. In this paper, we computationally evaluated and reported the inhibitory activity found in meridianins A–G, a group of marine indole alkaloids isolated from the marine tunicate *Aplidium*, against various protein kinases involved in Alzheimer’s disease (AD), a neurodegenerative pathology characterized by the presence of neurofibrillary tangles (NFT). Balance splitting between tau kinase and phosphate activities caused tau hyperphosphorylation and, thereby, its aggregation and NTF formation. Inhibition of specific kinases involved in its phosphorylation pathway could be one of the key strategies to reverse tau hyperphosphorylation and would represent an approach to develop drugs to palliate AD symptoms. Meridianins bind to the adenosine triphosphate (ATP) binding site of certain protein kinases, acting as ATP competitive inhibitors. These compounds show very promising scaffolds to design new drugs against AD, which could act over tau protein kinases Glycogen synthetase kinase-3 Beta (GSK3β) and Casein kinase 1 delta (CK1δ, CK1D or KC1D), and dual specificity kinases as dual specificity tyrosine phosphorylation regulated kinase 1 (DYRK1A) and cdc2-like kinases (CLK1). This work is aimed to highlight the role of CADD techniques in marine drug discovery and to provide precise information regarding the binding mode and strength of meridianins against several protein kinases that could help in the future development of anti-AD drugs.

## 1. Introduction

Drug discovery is the process of identifying new molecules with a certain therapeutic activity. This process is very expensive in terms of money and time. Translating basic research to the market (going through drug discovery, preclinical and clinical studies) takes tens of years and costs billions of dollars. The average cost to develop a new molecular entity is estimated to be $1.8 billion and requires about 13.5 years [1]. However, the usage of computational techniques at various stages of the drug discovery process could reduce that cost [2]. Hence, computer-aided drug discovery/design (CADD) methods are becoming very popular and during the last three decades have played a major role in the development of therapeutically important molecules [3,4]. CADD techniques cover several aspects of the drug discovery pipeline, ranging from the selection of candidate molecules to the optimization of lead compounds. For instance, virtual profiling (VP) methods can predict the biological profile as well as mechanisms of action (MoA) of a certain molecule; molecular modelling techniques, such as docking and molecular dynamics (MD), can predict ligand–target interactions in terms of binding mode and/or binding strength, allowing discrimination between candidate compounds [5,6]; virtual screening (VS) methods are able to find analogues (similar molecules) for a given compound(s) and/or build compound libraries from an input molecule(s); hit to lead (H2L) optimization techniques are used to design new molecules, improving an existing compound; absorption, distribution, metabolism, excretion and toxicity (ADMET) prediction techniques are able to predict the physicochemical properties of a given compound, i.e., information that can be coupled to H2L techniques in order to design better and safer drugs before synthetizing them. 

**Figure 1:**
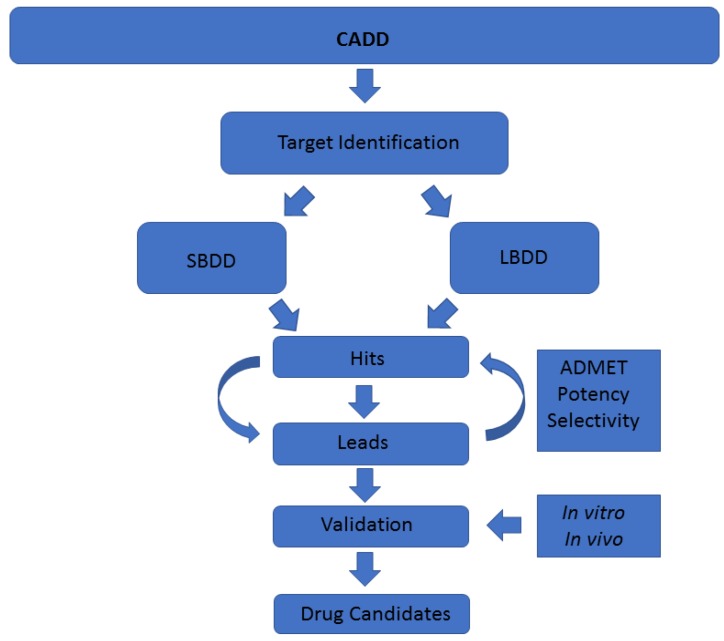
Schematic representation of the computer-aided drug discovery/design (CADD) techniques depicting a drug discovery pipeline.

A common classification of these techniques is based on the nature of the input molecule. In this sense, there are two general types of CADD approaches: structure-based drug design (SBDD) and ligand-based drug design (LBDD). In SBDD, macromolecular three-dimensional (3D) target structures, usually proteins, are analysed with the aim of identifying compounds that could interact (block, inhibit or activate) with them. In LBDD, chemical compounds are analysed in order to, for instance, find chemical analogues, explore their biological and/or toxicological profile, or improve their physicochemical and pharmacological characteristics with the aim of developing drug-like compounds (Figure 1) [7,8]. 

Historically, most new drugs have been designed from natural products (secondary metabolites) and/or from compounds derived from them [9]. Natural products have thus been a rich source of compounds for drug discovery, and often, feature biologically relevant molecular scaffolds and pharmacophore patterns that have evolved as preferred ligand–protein binding motifs. The United States Food and Drug Administration (US FDA) revealed that between 1981 and 2010, 34% of those medicines approved were based on small molecules from natural products or direct derivates of them [10,11]. The identification of natural products that are capable of modulating protein functions in pathogenesis-related pathways is one of the most promising lines followed in drug discovery [12]. Therefore, natural products constitute a huge source of inspiration in drug design [13].

An example is Alzheimer’s disease (AD), a neurodegenerative pathology that constitutes the most common type of dementia (60–80% of the total cases), characterized by the presence of neurofibrillary tangles (NFT) primarily composed of abnormal phosphorylated tau and senile plaques (SP). Nowadays, despite its high incidence, there is still no specific treatment approved to cure this disease. Tau phosphorylation is regulated by a balance between tau kinase and phosphate activities. Splitting of this balance was considered to cause tau hyperphosphorylation and thereby its aggregation and NTF formation [14,15]. Due to that fact, inhibition of specific tau kinases or kinases involved in tau phosphorylation pathway, could be one of the key strategies to reverse tau phosphorylation and, ultimately, fight AD [16]. 

The main relevant protein kinases involved in tau phosphorylation have been grouped into two classes: tau protein kinases and dual specificity kinases. The first group contains proteins such as glycogen synthetase kinase-3 beta (GSK3β), that phosphorylates tau at different sites (specifically at 42 sites, 29 of them phosphorylated in AD brains) and casein kinase 1 delta (CK1δ), a non-proline-directed protein kinase (non-PDPK) that regulates the microtubule dynamics through tau phosphorylation at 46 sites (25 of them phosphorylated in AD brains). The second group contains proteins such as dual specificity tyrosine phosphorylation regulated kinase 1 (DYRK1) that self-catalyse their autophosphorylation and behave as serine/threonine kinase that phosphorylates tau and the transcription factor cyclic adenosine monophosphate-response element binding (cAMP-CREB), and an evolutionarily conserved group of dual specificity kinases cdc2-like kinases (CLKs), which play an important role in the regulation of ribonucleic acid RNA splicing and are involved in the pathology of AD by phosphorylating the serine residues in arginine-rich (SR) proteins [14,15,17,18,19].

Among natural products, those of unexplored marine world origin are of great interest in the discovery of novel chemical structures, since they harbour most of the biodiversity of the world [20,21]. For instance, compounds from marine invertebrates may possess interesting pharmacological activities. Examples include Porifera, Cnidaria, Bryozoa, Mollusca and Tunicata [22,23]. However, although very interesting and useful from a pharmacological point of view, obtaining these compounds is difficult, both from technical and biological points of view; technically, because specimens have to be collected by hand using scuba diving or by trawling (both expensive, logistically difficult, and time consuming), and biologically, due to their marine habitats and due to the fact that they are usually unculturable [23]. All these factors, together with the adequate implementation of the Nagoya Protocol and the bioavailability of marine natural products, result in CADD contributions being highly relevant, since no biological sample is needed to perform an in silico analysis [24]. This also alleviates some of the marine drug discovery difficulties, such as the quantity of natural product necessary to be used in further clinical studies.

**Figure 2:**
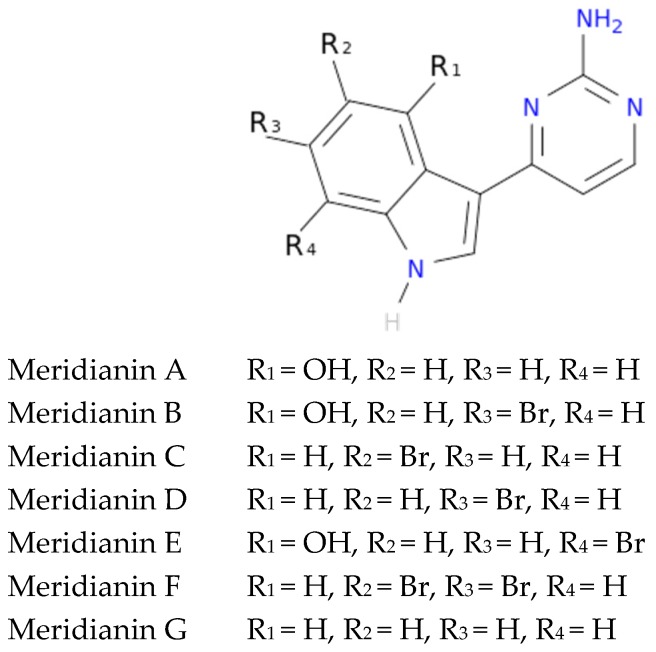
Structures of meridianins A–G.

To exemplify and highlight the power of CADD techniques in marine drug discovery, as part of an ongoing study of bioactive marine molecules from benthic invertebrates, in this paper we evaluated and reported the inhibitory activity found in meridianins A–G (Figure 2), a group of marine indole alkaloids consisting of an indole framework connected to an aminopyrimidine ring, isolated from specimens of the tunicate genus *Aplidium*, against various protein kinases involved in AD.

## 2. Results

### 2.1. Virtual Profiling

**Figure 3:**
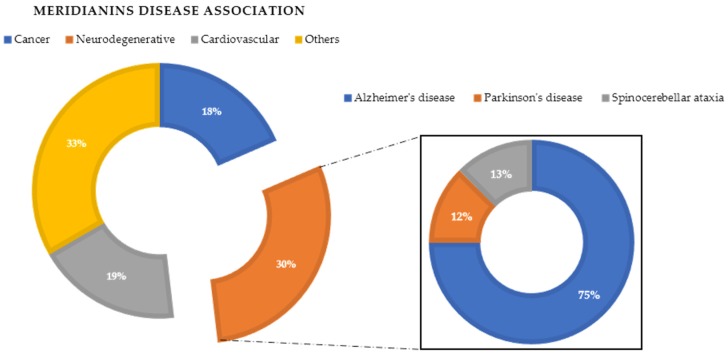
Disease association performed after a virtual profiling (VP) using meridianin A as a seed.

In a previous VP study (unpublished data not shown here), we observed that meridianins could bind to diverse targets involved in different diseases associated with aging or neurodegenerative pathologies, such as AD and Parkinson’s disease, cancer and cardiovascular diseases (Figure 3). The found targets are of special interest as they are involved in several diseases that affect millions of people worldwide, having a huge social incidence and also, in most cases, there is no cure for them. Regarding AD, the most common disease in which meridianins could have a therapeutic role according to our results (Figure 3), GSK3β, CK1δ, DYRK1A and CLK1 (four kinases involved in it) could be targeted. This finding can be easily checked in the literature, confirming that meridianins can bind to these kinases. Moreover, it can also be confirmed that the target analysis results are trust worthy, since not only is the involvement of meridianins in AD disease found in the literature, but the role of meridianins as anti-cancer agents can also be easily checked [25,26].

With these results in hand, the four kinases GSK3β, CK1δ, DYRK1A and CLK1 were selected for further analysis due to the prevalence of AD as the most common meridianin therapeutic target. 

### 2.2. Structural and Sequence Analysis

**Figure 4:**
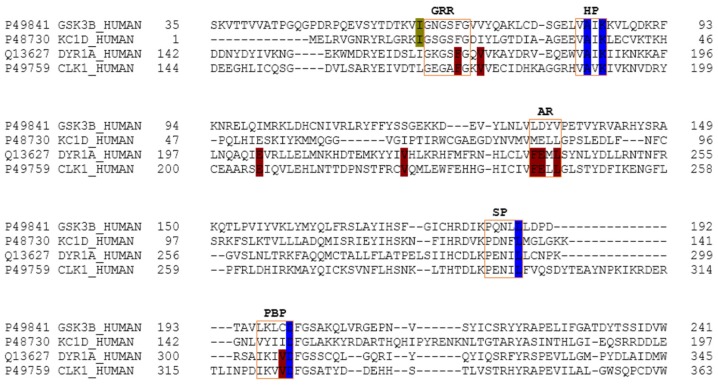
Amino acid sequence alignment of GSK3β, CK1δ, DYRK1A and CLK1. In the image, only the ATP-binding pocket residues are shown. In blue, the key residues are conserved between all kinases. Green shows those conserved residues between tau protein kinases GSK3β and CK1δ, and red shows those conserved in dual specificity kinase DYRK1A and CLK1. Key residues refer to the residues implied in the binding of all the meridianins shared by the different targets and that are evolutionary conserved. The orange boxes represent the diverse region of the adenosine triphosphate (ATP) binding pocket. GRR: glycine-rich region; HP: hydrophobic pocket; AR: adenine region; SP: sugar pocket; PBP: phosphate binding pocket.

Despite that the structural catalytic domains of most kinases are highly conserved, sequence alignment studies revealed some differences (Figure 4). The kinase catalytic domain, referred to as the hinge region, is divided into two lobes; the N-terminal mostly consists of β-sheets, whereas the C-terminal lobe is mainly helical. According to various authors, the adenosine triphosphate (ATP)-binding pocket of protein kinases can be divided into five regions: adenine region, sugar pocket, hydrophobic regions (I and II) and the phosphate-binding region [27,28,29]. In addition to this division, some recent studies have identified another important region: the glycine-rich loop, which is defined by the GxGxxG motif and is highly conserved among the protein kinase family. This region is suggested to significantly contribute to the potency and selectivity of binding inhibitors [29,30,31]. The glycine-rich loop and the hydrophobic pocket are placed in the so-called N-terminal region, while the sugar pocket and phosphate-binding region are located over the so-called C-terminal region. The adenine region is situated in the middle of these regions. We have found that meridianins are able to bind to all these regions, with a different binding strength depending on their chemical structure. 

As explained above, we analysed two classes of protein kinases, specifically four members of them. The core catalytic regions are conserved among all as they belong to the same enzyme subclass (EC 2.7) and protein family (protein kinase). However, this protein family is divided into subfamilies: serine-threonine protein kinases (EC 2.7.11), dual-specificity kinase (EC 2.7.12), protein-histidine kinases (EC 2.7.13) and other protein kinases (EC 2.7.99). Thus, it seems logical that the binding site may be more conserved among subfamilies, and even more so in lower classifications (sub-subfamilies such as tau protein kinases (EC 2.7.11.26) and dual specificity kinase (EC 2.7.12.1)) than among the whole family. Analysing our results, we have confirmed this trend. Several key residues (associated with the substrate/inhibitor binding mode and/or conforming the pocket(s)) are conserved between the four studied proteins (Figure 4), but a higher identity is observed by pairs. GSK3β and CK1δ share more catalytic residues between them than with DYRK1A and CLK1, and vice versa. This observation agrees with the finding of a common binding pattern between the four protein kinases plus another pattern per each subfamily.

### 2.3. In Silico Binding and Interaction Analysis

Meridianins bind to the ATP binding pocket of each of the selected targets, acting as ATP competitive inhibitors. Binding energies obtained after docking and MD simulations (summarized in Table A1) show a reasonably similar binding strength between the diverse meridianins and even among the four kinases. Despite that fact, it could be observed that meridianin F tends to show higher energies than the rest of the compounds. Moreover, in general, meridianins present better binding interaction energies against CK1δ, DYRK1A and CLK1 than GSK3β. It must be said that these differences are hardly noticeable and cannot constitute a unique and definitive prioritization tool.

The binding mode per meridianin and target (that slightly changes between each complex) is summarized in Table A2, Table A3, Table A4 and Table A5. Comparing the interacting residues with the identified binders (summarized in Table A6), it is clearly observed that meridianins could behave as inhibitors of the analysed kinases. Moreover, analysing the observed binding mode together with the identified binders and the conserved residues (Figure 4, Table A6 and Table A7), as mentioned above, some patterns of the general binding of meridianins to protein kinases could be extracted. It has to be highlighted that the majority of the residues found in these patterns are identified as binders. 

For tau protein kinases, GSK3β and CK1δ, 5 binding residues are shared between each of them, whereas for dual specificity kinases, DYRK1A and CLK1, 12 are conserved. Moreover, there are four residues conserved along the four analysed targets (Figure 4 and Table A7). Concretely, these residues are an alanine and a lysine placed in the hydrophobic pocket, a leucine in the sugar pocket and an aspartic acid in the phosphate binding region. Regarding tau protein kinases, there is also an isoleucine shared by GSK3β and CK1δ. In the case of dual specificity kinases, there are eight other shared binders, specifically, two phenylalanine, three valines, two glutamic acids and one leucine conserved and identified as binders. Analysing the meridianin binding mode by focusing on the conserved amino acids also identified as binders, we have found that two of them, A83 and K85 placed in the hydrophobic pockets, are present in all meridianin binding modes over GSK3β and CK1δ (in the latter case, numbered A36 and K38). For DYRK1A, three of the conserved residues are identified as key residues for the binding of all meridianins, specifically V173, L241 and L294, in the same way as for CLK1 (in this case numbering as V175, L244 and L295). In addition to these residues, others were found implicated in the general binding of meridianins not conserved through all the targets (Table A6), specifically, for GSK3β I62, V70, L132 and D200, for CK1δ I23, M82, L85, L135 and I148, and for DYRK1A K188, V222, F238, V306 and D307. Finally, CLK1 residues L167 and A189 were identified as key meridianin binders.

Besides the above-mentioned residues, there are other important residues per meridianin and target not present in the observed patterns that have a key role (Table A7), not dependent in a general behaviour but dependent on the particular nature of each meridianin and target (Table A2, Table A3, Table A4 and Table A5).

#### 2.3.1. Glycogen Synthetase Kinase-3 Beta

**Figure 5:**
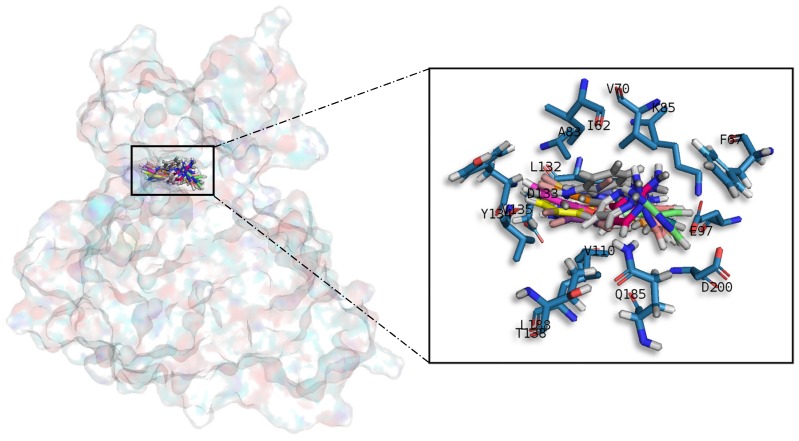
Meridianins A–G superposition over GSK3β. Labelled ligand-active site amino acid residues involved in binding and the binding position of each meridianin models are enlarged.

Meridianins (Figure 5) tend to be placed within adenine (LDYV motif) and the hydrophobic regions, formed by the conserved residues A83 and K85, in the catalytic cleft. The indole scaffold of the meridianins is wrapped by N-terminal I62, F67, V70, A83, K85 and C-terminal T138, Q185, L188, D200 residues together with the LDYV motif in the hinge adenine region. Core interaction residues stabilize meridianins by establishing hydrophobic contacts with I62, V70, A83, K85, L132, D200 and hydrogen bonds with I62, K85, D200. The observed results further suggest that meridianins establish interactions over the glycine-rich loop on GSK3β, defined by the GNGSFG motif, as well as with D200, a residue present in the phosphate pocket. The fact that meridianins bind to I62, V70, A83, K85, L132, L188 and D200, previously identified as binders, highlights meridianins inhibitory nature against GSK3β.

#### 2.3.2. Casein Kinase 1 Delta 

**Figure 6:**
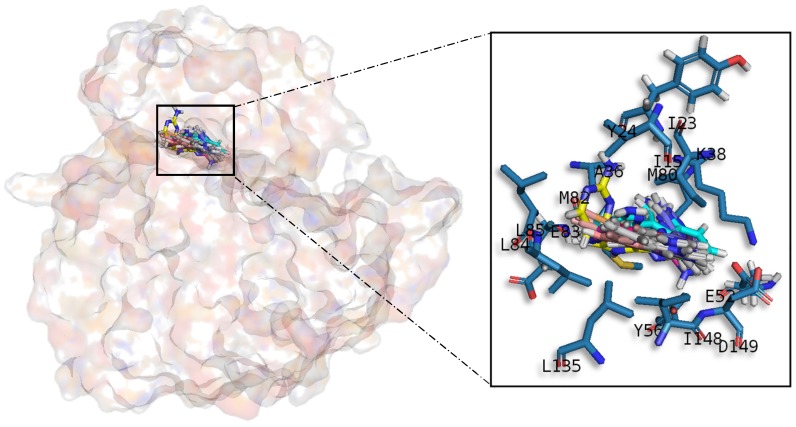
Meridianins A–G superposition over CK1δ. Labelled ligand-active site amino acid residues involved in binding and the binding position of each meridianin model are enlarged.

All meridianin structures (Figure 6) share common interactions occupying the adenine region formed by the MELL motif. Meridianins are stabilized in the hinge catalytic region, establishing hydrogen bonds with A36, K38, M82, L85 and hydrophobic contacts with I23, K38, M82, L85, L135, and I148. Interestingly, it has also been observed that the indole group of the higher ranked poses has additional interactions with N-terminal I15, Y24, A36 and C-terminal D149 residues. It is important to remark that meridianins bind to the previously identified binder residues I23, A36, K38, M82, L85, L135 and I148, a fact that highlights meridianins inhibitory nature against CK1δ.

#### 2.3.3. Dual Specificity Tyrosine Phosphorylation Regulated Kinase 1

**Figure 7:**
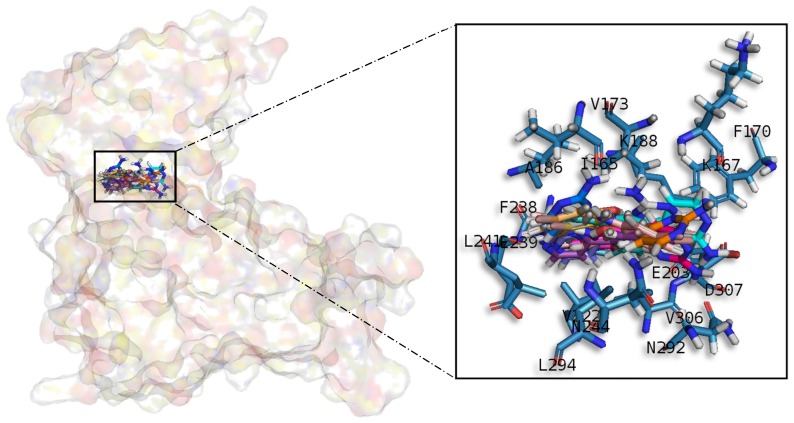
Meridianins A–G superposition over DYRK1A. Labelled ligand-active site amino acid residues involved in binding and the binding position of each meridianin model are enlarged.

Meridianins are placed on the C-terminal region over the phosphate and sugar pockets as well as the adenine motif FEML (Figure 7). Despite the fact that meridianins seem to interact with the N-terminal residue V173 and the hydrophobic pocket residue K188, the rest of the key interactions are established with residues placed over the C-terminal side. Meridianins establish hydrogen bonds with K188, L241 and V307 as well as hydrophobic contacts with V173, K188, V222, F238, L241, L294, V306 and V307. Moreover, they perform π–cation and π–π stacking interactions with F238, which belongs to the adenine motif. The inhibitory effect of meridianins against DYRK1A is confirmed by the fact that all of them bind to V173, K188, V222, F238, L241, L294, V306 and V307, i.e., residues previously identified as binders.

#### 2.3.4. Cdc2-Like Kinases

**Figure 8:**
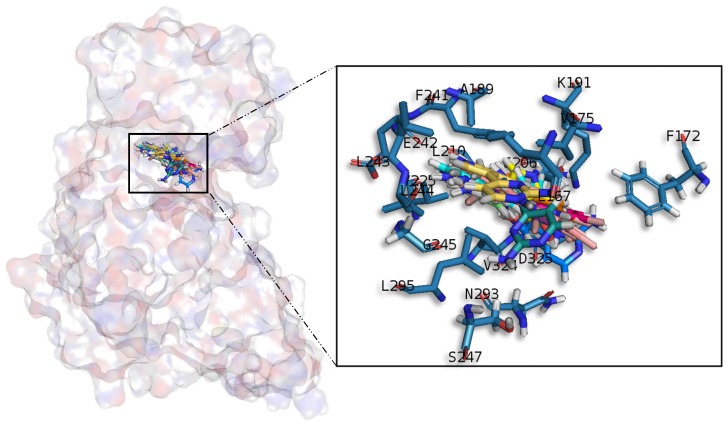
Meridianins A–G superposition over CLK1. Labelled ligand-active site aminoacid residues involved in binding and the binding position of each meridianin model are enlarged.

Meridianin A–G conformations against CLK1 differ by pose, as can be observed in the superimposition shown below (Figure 8). In fact, over this target is where meridianins displayed a more different conformation between the family members. In general, all poses tend to be located near the glycine rich loop and the hydrophobic pocket, interacting with the adenine motif FELL through L244 by a hydrogen bond interaction. The different poses were well stabilized into the hinge catalytic pocket by establishing hydrogen bonds interactions with L167 and L244 and hydrophobic contacts with L167, V175, A189, L244 and L295, all of them previously identified as binders, a fact that underline their inhibitory nature against CLK1.

### 2.4. Selectivity

Since the results of in silico binding showed good interactions against the four studied targets, we wanted to know whether meridianins could be selective inhibitors of the studied protein families. Thus, we conducted a selectivity test consisting of analysing the meridianins binding over eight kinases (seven protein kinases and one non-protein kinase) with the aim of observing meridianin binding preference. This test included meridianins and three compounds derived from them, previously proposed as kinase inhibitors with a good selectivity for DYRK1A and CLK1 [25,32]. Our results show that meridianins and the derived compounds are able to bind to all the studied protein kinases, suggesting that they are not selective among them, although, for isocitrate dehydrogenase cytoplasmic (IDH1) and cGMP-dependent protein kinase 1 (PRKG1), slightly lower binding energies can be observed. Moreover, althought compunds 1-3 tend to better interact with DYRK1A and CLK1, large differences are not observed in binding affinity between meridianins and their derived compounds (Table A8). In that sense, the derived compounds show a selectivity for DYRK1A and CLK1 respect to GSK3β and CK1δ, but not to all the tested kinases. Together, our results revealed the necessity to increase the selectivity of the meridianins and their, herein analysed, derived compounds.

### 2.5. Pharmacokinetic Properties

Due to the importance of pharmacokinetics (PK) studies during drug discovery, the whole set of meridianins and the three meridianins derived compounds were analysed, studying the ADMET properties for each molecule (Table A9, Table A10 and Table A11).

#### 2.5.1. Absorption Properties

In Caco-2 permeability, two different models were used as in the first one (ML model), compounds **1** and **2** cannot be evaluated because they are out of the applicability domain (OAD). All the analysed molecules have high permeability according to our proprietary model; while using pkCSM meridianin G and compounds **2** and **3** show low permeability values, but they are almost considered as high (>0.9). LogS values confirm good solubility in water and good bioavailability for each compound. Intestinal absorption shows quite good percentages (absorbance >88%) for all the studied compounds, as molecules showing values lower than 30% would be considered to be poorly absorbed. Both the P-glycoprotein (Pgp) substrate and I/II inhibitor models show good concordance, and all of the studied molecules have been predicted to be Pgp substrates, and any of them could act as an inhibitor. The last absorption property studied was skin permeability, and results show values >−2.76, which means reasonable low skin permeability. 

#### 2.5.2. Distribution Properties

Log P values were calculated. The steady state volume of distribution (VDss) show by the studied molecules is low, as all are above 2.81 L/Kg, Log VDss > 0.45. For plasma protein binding (PPB) property, all the studied compounds have a probability of binding >90%. Blood-brain barrier (BBB) permeability results show poor permeability for all meridianins and the three derived compounds. Compounds with a blood-brain permeability-surface area product (logPS) >−2 are considered to penetrate the central nervous system (CNS), and in that sense, compounds **2** and **3** could be considered as penetrants as they show slightly better results, i.e., logPS values of −1.88 and −1.99, respectively. However, they are on the border, and the general tendency of all of them is to show poor penetration.

#### 2.5.3. Metabolism Properties

Cytochrome P450 interaction reveal that all the molecules in the studied sets are likely to be metabolised. All of the analysed compounds are able to inhibit the CYP1A2 isoform. Besides meridianin F and compounds **1** and **2** can also inhibit the CYP2C19 isoform, and compounds **1** and **3** the CYP3A4 isoform. Moreover, compound **2** can act as a substrate of the CYP3A4 isoform (Table A11).

#### 2.5.4. Excretion Properties

None of the analysed compounds is a substrate of organic cation transporter 2 (OCT2), which means that non-clearance problems and adverse interactions with co-administrated OCT2 inhibitors are expected. Moreover, total clearance was measured.

#### 2.5.5. Toxicology Properties

Regarding the maximum recommended tolerated dose (MRTD), our results show that only meridianins A, B and E have high (greater than 0.477 log (mg/kg/day) MRTD values, which means that a higher dose could be administrated, while the other compounds show lower values. AMES toxicity predicts mutagenic and carcinogenic characteristics and the results reveal that only meridianins A, B and E have no apparent toxicity. The human ether-a-go-go gene (hERG) I and II inhibitor method show that any of the studied molecules is likely an hERG inhibitor. Hepatotoxicity results point out that meridianins B and F may be associated with disrupted normal function of the liver. Skin sensitisation results show no adverse effects for dermally applied products. In summary, based on all analysed compounds, only meridianins A and E seem to be non-toxic and administrable with a possible high dose without presenting adverse toxic effects.

## 3. Discussion

CADD techniques have an enormous potential in drug discovery, especially when they originate from marine natural products, as they do not waste natural resources. As mentioned, there are numerous different methodologies enclosed within the term CADD [2,4]. Usually the methodology is chosen based on its applicability, advantages/drawbacks, previous studies in the field, and also the expertise of the authors. In that sense, general methods such as docking, MD or ligand similarity searches have been developed, as well as more specific techniques such as disease or target models [33,34,35,36,37,38,39,40,41,42,43,44]. Each technique requires a specific input and gives a specific output, aiming to solve one step of the drug discovery pipeline (Figure 1). However, although individual CADD methods can provide insight and solve many questions, their power is their strength when combined, as we show here. With the techniques employed in this study, we have mostly covered the drug discovery process able to be coped computationally. The methodologies we show in this work, as well as the way and the order in which we have used them, are addressed to cover a plausible general pipeline, which in our opinion is of general interest regarding marine molecules discovery. In previous years, many resources have been invested in biodiscovery (for instance, European funded projects such as PharmaSea, MaCuMBA, SeaBiotech, BlueGenics or MicroB3) and some lead compounds have been designed, but a lot of information remains stored [45,46,47,48,49]. Using CADD techniques, this information could be easily analysed and, potentially, employed to find drug candidates. In summary, we have shown how starting from a molecule, we were able to provide lead compounds (although in this case we provide insights to construct them instead of fully designed compounds) against a certain disease. In that sense, and as we have commented above, we exemplified the role of CADD tools applied to marine drug discovery in general, and in this particular case, analysing the role of meridianins in AD, even more specifically, against four protein kinases involved in its pathology. 

The four protein kinases studied here were previously described by other authors as meridianin targets [25,32,50,51]. This constitutes an excellent validation of our computational, blind, approach to identify the biological profile of meridianins. However, although in the literature the possible anti-AD activity of meridianins was reported and several compounds have been designed from them [25,32,50,51], several aspects have not been taken into account and analysed, from a target-based (structural) perspective, as we have done here.

A common observed feature of protein kinases inhibitors is that most of them usually interact with the phosphate binding groove, in the innermost part of the pocket. This is a rich polar region, with groups such as arginine or aspartate, that consequently can create hydrogen bonds with small molecules acting as inhibitors [52]. We observed that meridianins also show this trend, supporting their already mentioned general kinase inhibitory capacity. This, in addition to the fact that most of the meridianin binding residues are previously described as binders of known inhibitors, as well as the enzymatic assays that validated meridianin binding against the four studied kinases, also reinforce their tau protein and dual specificity kinase inhibitory capacity. As mentioned above, to exert this inhibitory capacity, meridianins show general binding trends against protein kinases in general and the studied targets in particular, but also specific features related to the nature of each of the targets. The understanding of these interactions (meridianin–target) and the identification of which of these characteristics are the most important to obtain good interactions is key in the design of meridianin-derived kinase inhibitors.

It was observed that for GSK3β, the best scored meridianins C, D, E, and F (Table A1) establish hydrophobic contacts within the aminopyrimidine ring, revealing that this scaffold could be important in having optimal interactions. This highlights the fact that the most important interactions between GSK3β and meridianins were on the glycine rich loop and the hydrophobic and phosphate pockets. For CK1δ, analysing our in silico binding results, we observed that for the best scored meridianins C, D and F (Table A1), it seems that to increase the affinity of the ligand on this receptor, the aminopyrimidine moiety should be oriented towards the top of the hydrophobic pocket at the N-terminal region. Also, key interactions were observed in the adenine and sugar-phosphate pockets. Regarding DYRK1A, meridianins mostly tend to be located over phosphate and sugar pockets as well as the adenine motif FEML rather than the glycine rich loop. Best scored meridianins B, C, E, and F (Table A1), share similar conformations but with different orientation with respect to the rest of the analysed meridianins, a fact that could be exploited for future developments together with meridianins preferential placement over the phosphate and sugar pocket. For CLK1, our molecular modelling studies have revealed that the best interacting meridianins B, C, D and F (Table A1) tend to be located near the glycine rich loop and the sugar pocket. 

In general, the orientation of meridianin indole scaffolds differs from one complex to another. Its preferential positioning is directed by hydrophobic interactions and steric effects, due to the aminopyrimidine ring position. In some models, it occupies hydrophobic region I, similar to many potent serine/threonine or tyrosine kinase inhibitors [27]. It must also be mentioned that for GSK3β and CLK1, the preferred meridianin binding zones were located over the glycine rich loop (N-terminal). Nevertheless, over CK1δ and DYRK1A, meridianins tend to be located over the sugar and phosphate region (both over the C-terminal region), correlating this fact with the slightly highest interacting energy observed after in silico binding experiments (Table A1). This could establish a new insight into future development of inhibitors. 

**Figure 9:**
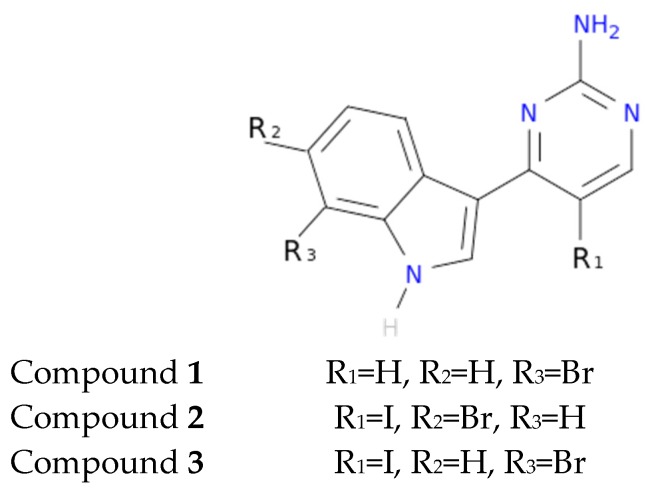
Structures of the three compounds. Selected from Bharate et al. Table 4—Compounds **68**–**70** and Giraud et al. Figure 2 —Compounds **30,33,34** [25,32].

Another interesting feature observed with respect to the meridianin binding mode is the presence of bromine. When present, interaction energies seem to be higher. The perfect example is meridianin F, which has two Br at R_2_ and R_3_, and has the best interaction energies for each of the studied targets with respect to the rest of meridianins. Emphasizing this issue, a pattern was observed within the two classes of kinases. For CK1δ meridianins C (Br = R_2_), D (Br = R_3_) and F present the best interaction energies. In GSK3β, meridianins D and F are among the three best interacting compounds. On DYRK1A, meridianins B (Br = R_3_), C and F are three of the four best interacting compounds and in CLK1, meridianins B, C, D and F are the ones that show the best energies. All these facts led us to hypothesize that Br on R_2_ and R_3_ on meridianins could be synonymous with potency and has to be taken into account for the design of new lead compounds against tau and dual-specificity kinases, in particular, and protein kinases in general. Interestingly, the most promising meridianin-derived compounds already designed (by Bharate and co-workers and Giraud and co-workers), are bromine-iodo derivates (compounds **2** and **3**) and noniodinated bromine analogues (compound **1**) (Figure 9) [25,32]. This fact supports our hypothesis about the influence of Br in the potency of binding showed by meridianins. According to our binding results, the derived compounds do not interact with target kinases stronger than do the meridianins. Therefore, we hypothesized that to design more potent inhibitors, the presence of Br atoms is key, but it is not enough. Playing with the different orientations and binding residues implicated in the observed patterns in meridianins-kinase binding should be also taken into account.

As protein kinases are a wide family of proteins involved in many cellular events, being selective against the desired ones is key, probably even more important than having a potent inhibitor, to avoid undesired effects. In that sense, our results show that both meridianins and the compounds reported by Bharate and co-workers, as well as Giraud and co-workers, could bind to different protein kinases with a similar strength [25,32]. In addition to that, the reported selectivity of the derived compunds for DYRK1A and CLK1 respect to GSK3β and CK1δ is observed, but it is not extensible to all the tested kinases. Going deeply into the results (Table A8), it could be observed that for IDH1 and PRKG1, the binding energies are slightly lower in comparison with the other targets. This fact is very relevant and could be explained because IDH1 is not a protein kinase. We put it in the pool of tested targets to see if out of the studied family, some selectivity could be observed. Regarding PRKG1, despite that it is a protein kinase member, the employed 3D structure contains an amino acid sequence that does not cover the kinase region. It was included to see what happened if despite being a protein kinase family member, the catalytic hinge region was not present. These findings allowed us to hypothesize that, despite meridianins do not show specific selectivity against any of the protein kinases tested, they do have a preferred binding to protein kinases. Moreover, this study validates the hypothesis that meridianins can act as protein kinase inhibitors. However, the low selectivity observed with respect to meridianins indicate that none of them is selective enough to properly act as AD therapeutic agent, even if able to inhibit the desired kinases. Although they could be a good starting point to design new drugs against AD, their selectivity should still be improved. To achieve that improvement, the presence of Br atoms is not enough. A rational design based on the structural differences and binding patterns observed along all meridianins should be carried out to obtain selective compounds that could have options to become an anti-AD drug. In that sense, the analysed derived compounds constitute an excellent example of how to improve meridianins to become therapeutic agents, but a new design is needed to overcome broader selectivity issues.

Potency and selectivity are important characteristics of a drug, but fulfilling certain ADMET requirements is also very important. The characterization of ADMET for the molecules being pursued as potential drug candidates is essential, as clinical failures of about 50% of the drugs under investigation are due to their inadequate ADMET attributes. In this regard, we have analysed the behaviour of all the studied meridianins and also the three compounds designed by Giraud and co-workers to evalute if the implemented modifications improve the properties of the meridianins (Table A9, Table A10 and Table A11) [25,32].

Meridianins and the three derived compounds show a potentially high, oral and intestinal, absorbance as well as reasonable low skin permeability. Probably one of the most relevant findings is that any of the studied compounds is able to cross the BBB by itself, which is essential for a drug that should act in the brain. Good penetration was not shown in the CNS in general. In addition to CNS entrance, the Pgp that seems to play a role in amyloid beta (Aβ) transport across the BBB and its modulation (inhibition) has been designed as a mechanism to improve CNS pharmacotherapy [53,54,55,56]. Unfortunately, any of the studied compounds has been predicted as an inhibitor, but as a substrate, which reinforces their inability to cross the gate into the CNS. Also, in relation to distribution properties, high PPB probabilities were observed as well as a low VDss, which means these compounds will have a lot of difficulties in diffusing or traversing cell membranes.

These compounds are also able to interact with cytochrom P450, acting as inhibitors and even substrates of some isoforms, as described in the results. As it is well known that CYP450 drug metabolism can induce clinical effects, these properties should be carefully analysed in order to design lead compounds from the herein studied molecules [57]. Moreover, toxicology predictors show that the studied molecules tend to have bad toxic effects, except meridianins A and E, for which no toxicity was predicted and the maximum tolerated dose increases with respect to the rest of the studied compounds.

Together, the obtained results suggest the necessity of performing a H2L optimization, in order to improve the absorption, distribution, metabolism and toxicity of the studied compounds, as well as their selectivity, with the aim of obtaining lead compounds able to become effective anti-AD drugs.

## 4. Materials and Methods

### 4.1. Virtual Profiling

VP techniques are computational tools aimed to elucidate the biological profile of a given molecule, for instance, therapeutic indications or targets of a chemical compound could be estimated. These techniques can be ligand- or target-based. Ligand-based approaches are able to automatically evaluate very large libraries or databases of compounds containing diverse information, for example, compound–target-bioactivities associations, using a chemical structure as a seed. As a result, similar molecules (restricted by a cut-off) are found and for instance, plausible targets to the input molecule selected. In this study, meridianin A was used as a seed. To run LBDD experiments, Cabrakan and Hurakan (Mind the Byte SL, Barcelona, Spain) software tools were employed [58,59]. Cabrakan is a two-dimensional (2D) ligand-based VP tool that compares molecules, through the use of 2D fingerprints, over a reference database and the assignment of biological activity. It allows the identification of similar chemical compounds (analogues) to the input molecule. Hurakan is a three-dimensional (3D) VP tool that compares a query molecule with the structures present in a reference database using Comparative Molecular Similarity Indices Analysis (CoMSIA) fields on a 3D grid. Hurakan can compare molecules according to their relationship with their environment, thus obtaining biomimetic compounds with different chemical structures. ChEMBL, which contains around 1,300,000 chemical compounds with detailed information including target data, was employed as the reference database [60]. A target was counted once when it appeared as both 2D and 3D hit during ligand-based VP experiments.

Here, we have employed similarity search based techniques, as they are simple, fast and accurate. However, they have the limitation imposed by the reference database employed. If there are no similar molecules to the input compound in the database, no results will be returned. This limitation is shared with other LBDD techniques such as quantitative structure–activity relationship (QSAR) or quantitative structure–property relationship (QSPR). The choice of these software tools and not another ones is based basically on the deep knowledge we have about the algorithm, the database and their performance.

Target-based approaches are able to, through knowledge of the 3D structures, evaluate huge databases that contain cavity information of these structures and after a binding site identification, docking calculations can be performed. As a result, the binding energy of every possible interaction is returned, which allows the classification and prediction of the best targets. In this study, meridianin A was used as a seed. Ixchel (Mind the Byte SL, Barcelona, Spain) is a structure-based VP tool that performs docking calculations of a molecule (spatial data file (SDF) or simplified molecular input line entry specification (SMILE) file) against an in-house developed database comprising almost 9000 protein cavities (binding-sites) curated from Research Collaboratory for Structural Bioinformatics Protein Data Bank (RCSB PDB) according to UniProt Knowledgebase (UniProtKB) human entries [61,62,63]. 

To run target (or virtual) profiling experiments related to SBDD, docking is the most used technique. MD simulations or related techniques could be also employed, but they are much too computationally expensive for these kinds of techniques, with docking the preferred option. There are several variants of the docking techniques, but as we have commented for LBDD, the main limitation is the reference database. In our case, we have selected a technique whose algorithm is well known and it also incorporates a curated database of which we have a deep understanding. A deep knowledge of the employed techniques is basic and based on that, we have selected Ixchel to run our experiments.

### 4.2. Structure Modelling

The meridianin structures were modelled from the 2D chemical structure published by Núñez-Pons, Avila and co-workers [26]. The three meridianins derived compounds used for the selectivity test were modelled from Giraud and co-workers and Bharate et al. [25,32].

Prior to any calculation, all the structures of the selected targets, for the binding and the selectivity analysis, were modelled from their crystal structures available from the Protein Data Bank (RCSB PDB). All of them represent human targets. As obtaining good structures is crucial, the best 3D structures were selected; the structures and chains that cover the maximum amino acid region sequence, in general, and the binding region of each of the selected targets in particular.

GSK3β was modelled from the crystallographic 3D structure with a PDB ID 3PUP that contains the crystallographic ligand OS1. It is stored in the PDB database as a homodimer, but only chain B was considered for further studies since GSK3β biological assembly is in monomeric form [31]. CK1δ was modelled from the 3D crystallographic structure corresponding to the entry 4KBK that contains the crystallographic ligand 1QG. Only chain B, since it is naturally a monomer, was considered for further studies [64]. DYRK1A was modelled from the crystal 3D structure with a PDB ID 4AZE that contains the crystallographic ligand 3RA. In the PDB database, we found 3 chains (A, B and C), but only chain A was considered for further studies as DYRK1A biological assembly is in a monomeric form [52]. CLK1 was modelled from the crystallographic 3D structure with a PDB code 2VAG with V25 as a crystallographic ligand. As this protein is naturally a monomer, there is only one chain in the PDB database, so further studies were performed against chain A [52].

To test selectivity, for all the PDB crystallographic structures selected, chain A was used in all cases. Structures were modelled from their respective crystallographic 3D structure: Fibroblast growth factor receptor 1 (FGFR1); 1AGW containing SU2 as a ligand, cAMP-dependent protein kinase catalytic subunit alpha (PRKACA); 2GU8 containing 796 as a ligand, hexokinase-2 (HK2); 2NZT containing BG6 as a ligand, dual specificity mitogen-activated protein kinase 1 (MAP2K1); 3DY7 containing ATP as a ligand, phosphatidylinositol 4,5-bisphosphate 3-kinase catalytic subunit gamma isoform (PIK3CG); 3IBE containing L64 as a ligand, PRKG1; 3OCP containing CMP as a ligand, serine/threonine-protein kinase N1 (PKN1); 4OTI containing MI1 as a ligand and one non protein-kinase IDH1; 4I3K containing NDP as a ligand.

To test the binding of meridianins and their selectivity, molecular modelling experiments were performed using the 3D structural models of meridianins A–G, and the models generated from the crystallographic structures available in the PDB (PDB ID 3PUP, 4KBK, 4AZE and 2VAG, respectively) and the PDB ID structures 1AGW, 2GU8, 2NZT, 3DY7, 3IBE, 3OCP, 4OTI and 4I3K, respectively.

### 4.3. Docking Calculations

Docking calculations constitute a simulation method, which predicts the preferred orientation of one molecule (ligand) to a second (target). When only the movements of the first molecule are allowed, the docking is considered classical or rigid; when both molecules are allowed to move, docking is considered flexible. Generally, docking, without any other specification, refers to classical (rigid) docking [7]. Docking, in the context of small-molecule drug discovery, concerns the study of binding process of small molecules (ligands) and targets (proteins), i.e., a candidate binding mode (pose) is predicted when ligand and receptor bind to each other. Scoring functions allow us to classify and rank, based on their calculated binding energies, the most favourable pose. In that sense, flexible docking has advantages over the rigid version of the technique. The dynamics is an intrinsic characteristic of proteins, necessary to carry out any of their functions. Flexibility incorporation within the binding mode prediction is key to obtain results capable of being correlated with experimental data. However, not all are advantages, as the predicted binding energies could worsen. The inclusion of additional degrees of freedom to simulate protein flexibility could increase the difficulty of accurately predicting the free energy of binding. This complication could arise because more contributions to the free energy must be considered, for instance, the interaction between flexible residues and the core of the protein, and typically, these additional contributions also introduce additional inaccuracies [65]. 

Another option to add flexibility is the post-processing of docking results, which means, for instance, docking validation and/or refinement by MD simulations. Rigid docking can predict the optimal placement of a ligand within the binding site of a receptor, but not all the key interactions between the ligand and receptor are usually depicted accurately. Hence, MD simulations can optimize the predicted binding mode and also check the stability of the docked complex, as a bad docking pose will generate an unstable MD trajectory, during which the ligand could even leave the binding site [34,36]. In this study, we have employed a pipeline aimed to simulate a flexible docking protocol in a similar way to other studies reported in the literature, in that we post-processed the obtained docking poses [66]. We selected this approximation as this two-step protocol constitutes a (probably the most) practical and convenient approach to address the docking problem [67]. It is in general less computational expensive and provides the results that we need in an accurate way, comparable to “real” flexible docking methodologies (such as ensemble-based or flexible induced-fit docking). In general, using MD as a post-processing tool, a smaller fraction of the conformational space is usually covered, but without the several limitations that affect sampling and scoring algorithms for docking.

All docking calculations were performed using Itzamna and Kin software tools (Mind the Byte SL, Barcelona, Spain) [68,69] to perform classical and blind docking calculations, respectively. Itzamna is used to carry out docking calculations and needs the structure of the molecule to dock, as well as the cavity where it should be placed as an input. Kin is a software tool designed to perform blind docking calculations. It involves a cavity search and a (best) cavity selection prior to performing the binding calculation; a difference of Itzamna is that the docking cavity is given as an input to the calculation. When the employed crystal structures were co-crystallized with a ligand, the cavity defined by the ligand was employed. As mentioned above, the modelled structures of the meridianins and the selected targets were used. Two runs were carried out for each calculation to avoid false positives. 

Results obtained from docking calculations were ranked based on their calculated binding affinities, and the best poses summarized in Table A1 and Table A8.

### 4.4. Molecular Dynamics Simulations

One of the principal tools in the computational studies of biomolecules are MD simulations, a theoretical method for studying the physical movements of atoms and molecules. MD calculates the time dependent behaviour of a molecular system, which means that atoms and molecules are allowed to interact for a fix period of time, giving a view of the dynamic evolution of the system. 

Short (1 nanosecond (ns)) MD simulations were performed using NAMD program version 2.11 over the best-docked complexes, which were selected based on ΔG_bind_ [70]_._ The Amber ff99SB-ILDN and the General Amber Force Field (GAFF) set of parameters were employed for modelling receptors and ligands, respectively [71,72]. The election of these force-fields was based on the fact that both have been extensively tested, being two of the most used for protein and protein-ligand simulations [71,72,73,74]. It has been shown that ff99SB-ILDN correlates consistently well with experimental data, and the GAFF force-field can conveniently and quickly produce reasonable ligand (especially organic molecules) parameters. Moreover, as amber force-fields, both are compatible, giving combined satisfactory results in several studies. Ligand GAFF parameters were obtained using Antechamber, whereas the receptor structures were modelled using the leap module of Amber Tools [75,76]. Simulations were carried out in explicit solvent using the TIP3P water model with the imposition of periodic boundary conditions via a cubic box [77]. Electrostatic interactions were calculated by the particle-mesh Ewald method using constant pressure and temperature conditions. Each complex was solvated with a minimum distance of 10 Å from the surface of the complex to the edge of the box. Temperature was kept at 300 Kelvin (K) using a Langevin Piston barostat. The time step employed was 2 femtoseconds (fs). Bond lengths to hydrogens were constrained with the SHAKE algorithm [78]. Before production runs, the system was energy minimized. Next, the solvent surrounding the protein was equilibrated at the target temperature using harmonic position restraints on the heavy atoms. Finally, the system was submitted to a slow heating-up phase, from 0 to 300 K. For the production run, all position restraints were removed. 

### 4.5. Molecular Mechanics/Generalized Born Surface Area (MM/GBSA)

The so-called reweighting techniques are computational approaches to estimate the alchemical free energy of interaction (ΔG_bind_) between small ligands and biological macromolecules. In the literature, MM/GBSA is usually employed to estimate ligand-binding affinities based on docking or MD simulations to get a more realistic view of the interaction of docked complexes. The obtained energies are more realistic than the docking interaction values, allowing a better ranking of the analysed compounds, although they cannot be biologically comparable. In our case and following similar approaches, we applied reweighting techniques, specifically MM/GBSA, over the generated MD trajectories for post-processing docking results [34,66,79].

MM/GBSA rescoring was performed using the MMPBSA python algorithm contained within the Amber Tools suite [80]. The snapshots generated in the 1 ns MD simulation were imputed into the post-simulation MM/GBSA calculation of binding free energy. MM/GBSA was chosen over other techniques such as molecular mechanics/Poisson–Boltzmann surface area (MM/PBSA), linear interaction energy (LIE), thermodynamics integration (TI) or free energy perturbation (FEP) because of its good balance between accuracy and computational cost.

Rigorous thermodynamic pathway approaches, such as TI or FEP, provide more accurate predicting binding free energies, whereas LIE, MM/GBSA and MM/PBSA constitute the so-called end-point methods that in general are less accurate. Each of these methods has its own strengths and limitations, and their computational requirements and speed are inversely correlated with their accuracy. TI and FEP, which outperform end-point approaches, are very useful, especially for ranking molecules inside a chemical series. Consequently, and regardless of their computational cost but given the computational advances, these techniques are gradually being more frequently used in the drug discovery pipeline, especially in guiding lead optimisation. However, in this study, our aim is not to provide a detailed library of lead compounds, and thus we have employed a less rigorous, but very popular approach in SBDD, alternative as the MMGBSA approach. The main problem of these techniques could be that the efficacy of the method is usually system dependent. However, it is generally accepted that they outperform docking results, so a better ranking of the analysed compounds will be always obtained, although, as commented above, the obtained binding energies could be far from being experimentally comparable.

### 4.6. Interaction Analysis

To analyse the key residues of the active site involved in the inhibitor binding, we examined the obtained binding modes after molecular modelling studies with already known binders of each of the targets. These binders (residues that have been revealed as necessary for the binding of known substrates/inhibitors) were identified through an evidence-based interaction analysis. It was carried out through a bibliographical search plus a database analysis. The bibliographical search was conducted using several studies in which inhibitors against the selected kinases were identified describing each compound binding mode [25,31,32,50,51,52,64,81,82]. The database search was done using an in-house, recently constructed database. It was built by crossing ChEMBL and the RCSB PDB [62], and it contains all PDB structures per UniProtKB ID with active compounds (by now there are only PDBS with compounds not competing against cofactors). Moreover, the database also contains the residues to which each active compound (per PDB) is bound. Thus, it allows the user, after docking or an MD calculation, to easily check whether the analysed molecules behave as a binder.

### 4.7. Sequence Analysis

The four targets were aligned using the UniProtKB clustal omega interface from the amino acid sequence associated with each UniProtKB entry.

### 4.8. Selectivity Analysis

Docking calculations of meridianins, as well as the three selected compounds (derived from them and described in the literature), against twelve protein kinases were performed. These meridianins derived molecules were obtained from the papers of Bharate et al. and Giraud et al. [25,32], and have shown interesting inhibitory concentration (IC_50_) values in the micro and sub-micromolar range, and a good selectivity for DYRK1A and CLK1. We selected them to see how the selectivity was taken into account in the design of these compounds as they strongly resemble the original meridianins scaffolds that we suspect are not selective enough.

To test the selectivity, we choose seven protein kinases, specifically, FGFR1, PRKACA, HK2, MAP2K1, PIK3CG, PRKG1 (for which the selected crystal structure do not contain the catalytic hinge), PKN1 and one non-protein kinase, IDH1. Thus, we tested if the selected compounds are selective between different protein kinases, belonging to different subfamilies, and between protein and non-protein kinases. Moreover, we explored if without the catalytic hinge, binding could be produced. 

### 4.9. ADMET Properties Prediction

For the meridianins and the three derived compounds, ADMET properties prediction was carried out using proprietary machine-learning (ML) models and the pkCSM webserver [83,84]. The proprietary ML models covered logS (molecular aqueous coefficient), logP (octanol/water partition coefficient), Caco2 permeability, BBB penetration and PPB. The first two models were generated by super vector regression (SVR) techniques and the last three employed supper vector machines (SVM). For training and testing the models, Chembl (logS, logP, Caco2) and Huuskonen (logS) datasets were employed, and for BBB and PPB, the datasets described by Muehlbacher et al. and Zhu and coworkers [85,86,87]. The pkCSM webserver allows the prediction of PK properties based on (I) compound general properties (including molecular properties, toxicophores and pharmacophore) and (II) distance-based graph signatures. Given an input molecule, both sources of information are used to train and test machine learning-based predictors. The webserver is composed of 28 (not all employed in this work) regression and classification ML models that have been generated and trained against 30 datasets (described at Pires et al.) [84].

The use of proprietary models, some of which are also covered by pkCSM, is because these methods, similar to other such as VS or VP, strongly rely on the employed reference dataset. As we have a deeper knowledge of our methods, we prefer to use them when possible. Only for Caco2 did we employ both models, ours and the pkCSM model, because for two compounds, our model is not good enough to make a reliable prediction (they are out of the applicability domain as they are too different with respect to the molecular fragments contained in the dataset employed to generate and train the model. If less than 90% of the molecular fragments in that the input molecule can be discomposed are not in the database, the prediction is not done). pkCSM predicted properties for all the compounds; however, it does not indicate if a prediction is out of the applicability domain.

In summary, we have analysed 21 ADMET properties, 5 of which were studied with our proprietary ML models and 17 with pkCSM. One of these properties, Caco2, was analysed twice using both our proprietary model and the pkCSM model.

#### 4.9.1. Absorption Properties

Caco2 permeability, LogS, intestinal absorption (human), P-glycoprotein substrate, P-glycoprotein I/II inhibitor and skin permeability. Caco-2 permeability is used to predict the absorption of orally administered drugs. A high permeability is assessed when the predicted valued is >0.90 for the pkCSM model, or high (H), in the proprietary model. LogS reflects the solubility of the molecule in water at 25 °C and also reflects the bioavailability of a given compound; it is represented by the logarithm of the molar concentration (log mol/L). Intestinal absorption indicates the portion of compounds absorbed through the human intestine; a molecule with an absorbance (intestinal absorption) of less than 30% is considered to be poorly absorbed. Pgp acts as a biological barrier by extruding toxins and xenobiotics out of cells, although it could have other, transport mediated, functions in certain tissues and organs. The predictor assesses whether a given compound is likely to be a substrate of Pgp. Pgp I and II inhibitors have significant PK implications for Pgp substrate, and the predictor will determine the inhibitory effect of a given compound against Pgp I/II, which could have advantages that can be exploited therapeutically, or result in contraindications. Skin permeability predicts if a given compound is likely to be skin permeable (logKp > −2.5).

#### 4.9.2. Distribution

LogP, VDss, PPB, BBB and CNS permeability. LogP allows us to estimate the distribution of a drug within the body (lipophilicity). VDss, which is the theoretical volume that the total dose of a drug would need to be uniformly distributed to give the same concentration as in blood and plasma, is considered low if log VDss <−0.15 and high if >0.45 (the higher the VD, the greater the drug distribution in tissue rather than plasma). PPB estimates the probability (>90% is considered high) that a given molecule binds to a plasma protein, the less bound a drug is, the more efficiently it can traverse cell membranes or diffuse. BBB permeability describes the ability of a drug to cross into the brain. The predictor describes whether a compound is able to cross the BBB. CNS permeability measures blood brain permeability surface-area (logPS), and it is similar to BBB but more direct, as it lacks the systemic distribution effects that may distort brain penetration. Compounds with a logPS >−2 are considered to penetrate CNS, while those with logPS <−3 are considered unable to penetrate.

#### 4.9.3. Metabolism

CYP450. Cytochrom P450 isoforms are important detoxification enzymes in the body and are essential for the metabolism of many medications. Drugs can be inhibitors of CYP450, blocking its metabolic activity, or can be metabolised (substrate) by them. CYP metabolism predictor assess whether a given molecule is likely to be metabolised or not and act as inhibitor of specific isoforms of CYP450; a specific inhibitor of CYP1A2, CYP2C19, CYP2C9, CYP2D6 and CYP3A4 and/or substrate of CYPD26 and CYP3A4.

#### 4.9.4. Excretion

Renal OCT2 substrate and Total Clearance. OCT2 is a renal uptake transporter that plays an important role in disposition and renal clearance of drugs and endogenous compounds. The OCT2 substrate predictor indicates if a given molecule is likely to be an OCT2 substrate, which provides not only clearance-related information but potential contraindications. Total clearance is related to bioavailability and is also important for determining dosing rates to achieve steady-state concentrations, and the predictor measures their value in log(mL/min/kg).

#### 4.9.5. Toxicology

MRTD, AMES toxicity, hepatotoxicity, skin sensitization, hERG I/II inhibitors. MRTD provides an estimated of the toxic dose threshold of chemicals in humans, and results less than or equal to 0.477 log(mg/kg/day) are considered low, and high when greater than 0.477 log(mg/kg/day). AMES toxicity indicates if a compound could be mutagenic and therefore may act as a carcinogen. hERG I and II inhibitor predictors determine if a given compound is likely to be a hERG I/II inhibitor as the inhibition of potassium channels encoded by hERG could result in fatal pathologies (for instance it is the principal cause of the development of acquiring long QT syndrome, fatal arrhythmia) and the withdrawal of many substances from the pharmaceutical market. Hepatotoxicity predicts if a given molecule is likely to be associated with disrupted normal function of the liver. Skin permeability predicts if a given compound is likely to be associated with skin sensitisation.

### 4.10. Graphical Representations

Graphical representations of protein-ligand complexes were prepared using PyMOL version 1.7 [88] and PLIP version 1.3.0 [89].

## 5. Conclusions

Meridianins can be classified as kinase inhibitors and can be used as a starting point to design and develop novel anti-AD drugs. It has been demonstrated, in silico and in vitro, that they are able to bind specific tau (GSK3β and CK1δ) and dual-specificity (DYRK1A and CLK1) protein kinases. However, they are not selective enough to constitute a therapeutic treatment against AD by themselves. In fact, as they are demonstrated to be protein kinase inhibitors, they could probably inhibit several kinases involved in different diseases [90]. In any case, they could serve as a starting scaffold to design new anti-AD drugs. To achieve that, a rational design taking advantage of the differences found in the binding patterns against different protein-kinases subfamilies, has to be carried out. In that sense, the presence of Br on R_2_ and the R_3_ position over the meridianin indole scaffold could be synonymous with potency. Besides, it seems that exploiting the C-terminal region (sugar and phosphate pocket) rather than the N-terminal side, could increase the strength of the interactions exerted by meridianins, and probably the potency shown by the designed compounds. However, although potency is important, and maintaining the presence of Br seems to be fairly accomplished [25,32], the selectivity between protein-kinase subfamilies is a crucial point to design proper anti-AD drugs, and even anti-cancer drugs. Meridianinsare not selective enough and should be improved to gain functionality and applicability. In addition, their measured ADMET properties indicate that they should be optimized in order to become a drug or at least a drug-lead compound. Therefore, the above-mentioned rational design in order to improve the potency and selectivity of meridianins should include H2L optimization cycles. The showed toxicity should be removed, and compounds interaction with Cytochrom P450 carefully analysed and, given the case, eliminated or modulated. Moreover, their distribution properties should be improved, lowering the PPB and VDss, to be able to diffuse and penetrate into cells easily. Besides, a mechanism to cross the BBB should be found and in that sense, modifying each compound to be Pgp inhibitors could be a possible strategy, although there are other mechanisms to overcome the BBB, including other protein binding and nanodelivery, that could be also exploited [91,92,93].

Regarding meridianins specifically and CADD methods in general, we can conclude that these techniques, despite their drawbacks, are very helpful in drug discovery, constituting a powerful tool that could save time and money in experiments. Our study with meridianins is an example of this, since we have been able to find plausible targets, that in the case of AD and cancer we have already validated through the literature. The key role that these techniques could have in drug discovery is even higher for the discovery and development of marine drugs, since no sample is needed to run these virtual experiments. Moreover, since these methods could point out the best direction to follow and in which targets expand the low sample amount that usually is available, these are crucial technologies to maximize the success of marine prospection, as well as to protect biodiversity.

## Appendix A

**Table A1:** Summary of classical rigid docking and Molecular Mechanics/Generalized Born Surface Area (MM/GBSA) calculations of the two best models selected per meridianins A–G. All energies values are kcal/mol.

	GSK3β			CK1δ			DYRK1A			CLK1	
	Binding Energy (kcal/mol)	MM/GBSA (kcal/mol)		Binding Energy (kcal/mol)	MM/GBSA (kcal/mol)		Binding Energy (kcal/mol)	MM/GBSA (kcal/mol)		Binding Energy (kcal/mol)	MM/GBSA (kcal/mol)
	R0/R1			R0/R1			R0/R1			R0/R1	
**A**	−7.3/−7.3	−26.43	**A**	−6.9/−6.9	−32.25	**A**	−7.4/−7.3	−28.00	**A**	−8.9/−8.9	−27.49
	−6.6/−6.1	−24.95		−6.8/−6.8	−30.10		−7.5/−7.4	−31.43		−7.8/−7.8	−30.70
**B**	−7.3/−7.2	−29.11	**B**	−6.4/−6.4	−35.06	**B**	−7.7/−6.9	−37.38	**B**	−8.5/−8.5	−34.14
	−6.8/−6.7	−29.25		−5.6/−5.5	−32.30		−7.3/−7.9	−34.03		−8.0/−8.0	−30.38
**C**	−7.6/−7.6	−28.54	**C**	−6.9/−6.9	−38.85	**C**	−8.2/−8.2	−31.95	**C**	−8.5/−8.5	−33.31
	−7.4/−7.5	−31.44		−6.9/−6.7	−35.84		−7.6/−7.6	−35.90		−8.1/−8.1	−34.92
**D**	−7.7/−7.7	−31.19	**D**	−7.0/−7.0	−38.69	**D**	−7.9/−7.9	−29.47	**D**	−8.6/−8.6	−33.58
	−7.0/−6.9	−30.01		−6.8/−6.6	−38.06		−7.5/−7.6	−34.59		−8.1/−8.1	−35.90
**E**	−7.3/−7.3	−31.26	**E**	−7.0/−7.0	−35.20	**E**	−7.5/−7.4	−35.62	**E**	−9.0/−8.8	−26.39
	−7.5/−7.5	−28.43		−7.0/−7.0	−34.97		−7.6/−7.4	−32.55		−7.9/−7.9	−31.63
**F**	−7.9/−7.9	−35.18	**F**	−7.2/−7.3	−38.55	**F**	−8.0/−7.8	−39.99	**F**	−8.7/−8.7	−37.71
	−7.7/−7.9	−34.73		−7.1/−7.1	−38.93		−7.8/−7.7	−39.91		−8.5/−8.5	−37.61
**G**	−7.3/−7.3	−24.04	**G**	−6.8/−6.8	−31.92	**G**	−8.1/−8.1	−30.17	**G**	−9.1/−9.1	−27.95
	−7.2/−7.2	−27.03		−6.9/−6.9	−32.94		−8.1/−8.1	−30.52		−8.7/−8.7	−29.88

To avoid false positives, each docking calculation was performed twice (R0 and R1).

**Table A2:** GSK3β residues that interacted with meridianins (each represented by letters A–G) after molecular dynamics (MD) simulations. Those residues involved in all meridianin binding are bold and were considered important binding residues.

Residues	A	B	C	D	E	F	G
**I62**	X	X	X	X	X	X	X
F67		X		X	X		X
**V70**	X	X	X	X	X	X	X
**A83**	X	X	X	X	X	X	X
**K85**	X	X	X	X	X	X	X
E97	X			X	X	X	X
V110	X	X	X		X	X	X
**L132**	X	X	X	X	X	X	X
D133	X			X		X	X
Y134	X	X	X	X		X	
V135	X	X	X		X		X
T138		X	X		X		
Q185				X			
L188	X	X	X	X	X	X	
**D200**	X	X	X	X	X	X	X

**Table A3:** CK1δ residues that interacted with meridianins (each represented by letters A–G) after MD simulations. Those residues involved in all meridianin binding are in bold and were considered important binding residues.

Residues	A	B	C	D	E	F	G
I15	X	X	X	X	X		
**I23**	X	X	X	X	X	X	X
Y24		X					
**A36**	X	X	X	X	X	X	X
**K38**	X	X	X	X	X	X	X
E52		X	X		X		
Y56	X			X		X	X
M80				X		X	X
**M82**	X	X	X	X	X	X	X
E83	X	X			X		
L84	X	X		X			
**L85**	X	X	X	X	X	X	X
**L135**	X	X	X	X	X	X	X
**I148**	X	X	X	X	X	X	X
D149	X	X	X	X	X		X

**Table A4:** DYRK1A residues that interacted with meridianins (each represented by letters A–G) after MD simulations. Those residues involved in all meridianin binding are in bold and were considered important binding residues.

Residues	A	B	C	D	E	F	G
I165	X	X		X	X	X	X
K167				X			
F170	X		X	X	X	X	
**V173**	X	X	X	X	X	X	X
A186	X	X	X		X	X	X
**K188**	X	X	X	X	X	X	X
E203							X
**V222**	X	X	X	X	X	X	X
**F238**	X	X	X	X	X	X	X
E239		X			X	X	X
**L241**	X	X	X	X	X	X	X
N244				X			
N292					X		
**L294**	X	X	X	X	X	X	X
**V306**	X	X	X	X	X	X	X
**D307**	X	X	X	X	X	X	X

**Table A5:** CLK1 residues that interacted with meridianins (each represented by letters A–G) after MD simulations. Those residues involved in all meridianin binding are in bold and were considered important binding residues.

Residues	A	B	C	D	E	F	G
**L167**	X	X	X	X	X	X	X
F172	X	X	X		X	X	
**V175**	X	X	X	X	X	X	X
**A189**	X	X	X	X	X	X	X
K191	X	X	X	X	X		X
E206			X				X
L210			X				
V225	X	X	X	X		X	X
F241	X	X	X	X		X	X
E242			X	X	X	X	
L243	X		X	X			
**L244**	X	X	X	X	X	X	X
G245					X		
S247					X		
N293							X
**L295**	X	X	X	X	X	X	X
V324	X	X	X	X	X		X
D325	X	X	X	X	X		X

**Table A6:** Binder columns represent those residues identified after a bibliographic and database research and that interacted with other inhibitors. In shared columns are those residues involved with all meridianins binding per target. Residue number corresponds to each Protein Data Bank (PDB) number.

GSK3β		CK1δ		DYRK1A		CLK1	
Binders	Shared	Binders	Shared	Binders	Shared	Binders	Shared
I62	I62					L167	L167
V70	V70	I23	I23	V173	V173	V175	V175
A83	A83	A36	A36			A189	A189
K85	K85	K38	K38	K188	K188	K191	
				V222	V222	V225	
L132	L132	M82	M82	F238	F238	F241	
V135		L85	L85	L241	L241	L244	L244
L188		L135	L135	L294	L294	L295	L295
		I148	I148	V306	V306	V324	
D200	D200	D149		D307	D307		

**Table A7:** Residues involved in all meridianins binding to GSK3β, CK1δ, DYRK1A and CLK1. Residue number corresponds to each PDB number.

GSK3β	CK1δ	DYRK1A	CLK1
I62	I15	I165	L167
		K167	
F67		F170	F172
V70	I23	V173	V175
	Y24		
A83	A36	A186	A189
K85	K38	K188	K191
E97			
	E52	E203	E206
	Y56		L210
	M80		
V110		V222	V225
L132	M82	F238	F241
D133	E83	E239	E242
Y134	L84		L243
V135	L85	L241	L244
T138		N244	G245
Q185			S247
		N292	N293
L188	L135	L294	L295
	I148	V306	V324
D200	D149	D307	D325

**Table A8:** Summary of classical rigid docking of the best model selected per meridianin A–G and the derived compounds **1**, **2** and **3**, against others protein kinases and one non-kinase (IDH1).

	**GSK3β**		**CK1δ**		**DYRK1A**		**CLK1**		**FGFR1**		**PRKACA**
	**Binding Energy**		**Binding Energy**		**Binding Energy**		**Binding Energy**		**Binding Energy**		**Binding Energy**
	**(kcal/mol)**		**(kcal/mol)**		**(kcal/mol)**		**(kcal/mol)**		**(kcal/mol)**		**(kcal/mol)**
	**R0/R1**		**R0/R1**		**R0/R1**		**R0/R1**		**R0/R1**		**R0/R1**
**A**	−7.3/−7.3	**A**	−6.9/−6.9	**A**	−7.4/−7.3	**A**	−8.9/−8.9	**A**	−7.1/−7.1	**A**	−8.5/−8.5
**B**	−7.3/−7.2	**B**	−6.4/−6.4	**B**	−7.7/−6.9	**B**	−8.5/−8.5	**B**	−6.7/−6.7	**B**	−8.5/−8.5
**C**	−7.6/−7.6	**C**	−6.9/−6.9	**C**	−8.2/−8.2	**C**	−8.5/−8.5	**C**	−7.1/−7.1	**C**	−9.0/−9.0
**D**	−7.7/−7.7	**D**	−7.0/−7.0	**D**	−7.9/−7.9	**D**	−8.6/−8.6	**D**	−7.1/−7.1	**D**	−8.8/−8.8
**E**	−7.3/−7.3	**E**	−7.0/−7.0	**E**	−7.5/−7.4	**E**	−9.0/−8.8	**E**	−7.4/−7.4	**E**	−7.6/−7.6
**F**	−7.9/−7.9	**F**	−7.2/−7.3	**F**	−8.0/−7.8	**F**	−8.7/−8.7	**F**	−7.3/−7.3	**F**	−8.5/−8.5
**G**	−7.3/−7.3	**G**	−6.8/−6.8	**G**	−8.1/−8.1	**G**	−9.1/−9.1	**G**	−7.1/−7.1	**G**	−8.6/−8.6
**1**	−7.6/−7.6	**1**	−7.0/−7.0	**1**	−8.1/−8.1	**1**	−9.2/−9.2	**1**	−7.1/−7.1	**1**	−8.2/−8.2
**2**	−7.7/−7.7	**2**	−7.1/−7.1	**2**	−8.2/−8.2	**2**	−7.4/−7.4	**2**	−6.2/−6.2	**2**	−8.3/−8.3
**3**	−8.0/−8.0	**3**	−7.3/−7.3	**3**	−7.9/−7.9	**3**	−7.8/−7.8	**3**	−6.4/−6.4	**3**	−8.0/−8.0
	**HK2**		**MAP2K1**		**PIK3CG**		**PRKG1**		**IDH1**		**PKN1**
	**Binding Energy**		**Binding Energy**		**Binding Energy**		**Binding Energy**		**Binding Energy**		**Binding Energy**
	**(kcal/mol)**		**(kcal/mol)**		**(kcal/mol)**		**(kcal/mol)**		**(kcal/mol)**		**(kcal/mol)**
	**R0/R1**		**R0/R1**		**R0/R1**		**R0/R1**		**R0/R1**		**R0/R1**
**A**	−7.1/−7.1	**A**	−7.6/−7.6	**A**	−6.8/−6.8	**A**	−6.3/−6.3	**A**	−5.8/−5.8	**A**	−7.8/−7.8
**B**	−6.6/−6.6	**B**	−7.4/−7.4	**B**	−7.3/−7.3	**B**	−6.5/−6.5	**B**	−6.3/−6.3	**B**	−7.7/−7.7
**C**	−7.0/−7.0	**C**	−7.2/−7.2	**C**	−7.0/−7.0	**C**	−6.6/−6.6	**C**	−5.8/−5.8	**C**	−8.1/−8.1
**D**	−6.6/−6.6	**D**	−7.4/−7.4	**D**	−7.8/−7.8	**D**	−6.9/−6.9	**D**	−5.8/−5.8	**D**	−7.3/−7.3
**E**	−6.8/−6.8	**E**	−6.7/−6.7	**E**	−7.1/−7.1	**E**	−6.3/−6.3	**E**	−5.6/−5.6	**E**	−7.2/−7.2
**F**	−6.9/−6.9	**F**	−7.5/−7.5	**F**	−7.2/−7.2	**F**	−6.8/−6.8	**F**	−6.0/−6.0	**F**	−7.8/−7.8
**G**	−6.9/−6.9	**G**	−7.5/−7.5	**G**	−7.3/−7.3	**G**	−6.5/−6.5	**G**	−6.3/−6.3	**G**	−7.9/−7.9
**1**	−6.9/−6.9	**1**	−7.4/−7.4	**1**	−7.7/−7.7	**1**	−6.4/−6.4	**1**	−5.8/−5.8	**1**	−7.4/−7.4
**2**	−8.1/−8.1	**2**	−7.5/−7.5	**2**	−7.3/−7.3	**2**	−5.3/−5.3	**2**	−6.1/−6.1	**2**	−7.7/−7.7
**3**	−7.1/−7.1	**3**	−7.2/−7.2	**3**	−7.3/−7.3	**3**	−6.1/−6.1	**3**	−6.0/−6.0	**3**	−7.7/−7.7

All energies values are kcal/mol. To avoid false positives, each docking calculation was performed twice (R0 and R1).

**Table A9:** Summary of ADMET properties of meridianins (A to G) and the derived compounds extracted from the literature (**1**–**3**).

**ABSORPTION**			**LogS**		**Caco2 Permeability**			**Caco2 * Permeability**			**Intestinal Absorption**		**Skin Permeability**
	**A**		−4.18	**A**	H	**A**		0.99	**A**		93.38%	**A**	−2.76
	**B**		−5.02	**B**	H	**B**		1.07	**B**		92.22%	**B**	−2.76
	**C**		−5.55	**C**	H	**C**		0.95	**C**		91.77%	**C**	−2.92
	**D**		−5.55	**D**	H	**D**		0.95	**D**		92.715	**D**	−2.91
	**E**		−5.04	**E**	H	**E**		0.98	**E**		90.98%	**E**	−2.74
	**F**		−6.16	**F**	H	**F**		0.98	**F**		91.49%	**F**	−2.92
	**G**		−4.51	**G**	H	**G**		0.86	**G**		93.44%	**G**	−2.90
	**1**		−4.18	**1**	OAD	**1**		0.93	**1**		91.41%	**1**	−2.90
	**2**		−5.02	**2**	OAD	**2**		0.8	**2**		89.89%	**2**	−2.884
	**3**		−5.55	**3**	H	**3**		0.819	**3**		91.04%	**3**	−2.895
		**P-Glycoprotein Substrate**			**P-Glycoprotein I/II Inhibitor**	**DISTRIBUTION**		**LogP**			**BBB**		**PPB**
**A**		Yes		**A**	No		**A**	1.53	**A**		No	**A**	>90%
**B**		Yes		**B**	No		**B**	2.39	**B**		No	**B**	>90%
**C**		Yes		**C**	No		**C**	3.10	**C**		No	**C**	>90%
**D**		Yes		**D**	No		**D**	3.10	**D**		No	**D**	>90%
**E**		Yes		**E**	No		**E**	2.40	**E**		No	**E**	>90%
**F**		Yes		**F**	No		**F**	3.58	**F**		No	**F**	>90%
**G**		Yes		**G**	No		**G**	2.44	**G**		No	**G**	<50%
**1**		Yes		**1**	No		**1**	3.40	**1**		No	**1**	>90%
**2**		Yes		**2**	No		**2**	3.40	**2**		No	**2**	>90%
**3**		Yes		**3**	No		**3**	3.10	**3**		No	**3**	>90%
		**VDss**			**CNS Permeability**	**METABOLISM**		**CYP450 Metabolism ***	**EXCRETION**		**Total Clearance**		**Renal OCT2 Substrate**
**A**		0.25		**A**	−2.92		**A**	Yes		**A**	0.57	**A**	No
**B**		0.24		**B**	−2.92		**B**	Yes		**B**	0.30	**B**	No
**C**		−0.06		**C**	−2.81		**C**	Yes		**C**	0.09	**C**	No
**D**		−0.01		**D**	−2.82		**D**	Yes		**D**	0.14	**D**	No
**E**		0.22		**E**	−2.93		**E**	Yes		**E**	0.15	**E**	No
**F**		0.07		**F**	−2.82		**F**	Yes		**F**	−0.19	**F**	No
**G**		−0.10		**G**	−2.12		**G**	Yes		**G**	0.71	**G**	No
**1**		−0.02		**1**	−2.83		**1**	Yes		**1**	−0.07	**1**	No
**2**		−0.09		**2**	−1.88		**2**	Yes		**2**	−0.092	**2**	No
**3**		−0.09		**3**	−1.99		**3**	Yes		**3**	0.132	**3**	No

Caco2 permeability is calculated using proprietary ML model and Caco2 * with the pkCSM webserver, as explained in the methods section. CCYP450 metabolism * specific values of interaction with different CYP450 isoforms are listed in Table A11. BBB: blood brain Barrier, PPB: protein-protein binding, VDss: steady state volume of distribution, CNS: central nervous system, OCT2: organic cation transported 2.

**Table A10:** Summary of toxicity properties of meridianins A–G and the three derived compounds extracted from the literatures (**1**–**3**).

	MRTD		AMES Toxicity		hERG I/II Inhibition		Hepatotoxicity		Skin Sensitisation
**A**	0.503	**A**	No	**A**	No	**A**	No	**A**	No
**B**	0.584	**B**	No	**B**	No	**B**	Yes	**B**	No
**C**	−0.107	**C**	Yes	**C**	No	**C**	No	**C**	No
**D**	−0.095	**D**	Yes	**D**	No	**D**	No	**D**	No
**E**	0.589	**E**	No	**E**	No	**E**	No	**E**	No
**F**	−0.088	**F**	Yes	**F**	No	**F**	Yes	**F**	No
**G**	−0.086	**G**	Yes	**G**	No	**G**	No	**G**	No
**1**	−0.068	**1**	Yes	**1**	No	**1**	No	**1**	No
**2**	−0.038	**2**	Yes	**2**	No	**2**	No	**2**	No
**3**	−0.058	**3**	Yes	**3**	No	**3**	No	**3**	No

MRTD: maximum recommended tolerated dose, hERG: human ether-a-go-go gene.

**Table A11:** Summary of specific values of interaction with different CYP450 isoforms properties of meridianins A–G and the three derived compounds (**1**–**3**).

CYP2D6 Substrate		CYP3A4 Substrate		CYP1A2 Inhibitor		CYP2C19 Inhibitor		CYP2C9 Inhibitor		CYP2D6 Inhibitor		CYP3A4 Inhibitor	
**A**	No	**A**	No	**A**	Yes	**A**	No	**A**	No	**A**	No	**A**	No
**B**	No	**B**	No	**B**	Yes	**B**	No	**B**	No	**B**	No	**B**	No
**C**	No	**C**	No	**C**	Yes	**C**	No	**C**	No	**C**	No	**C**	No
**D**	No	**D**	No	**D**	Yes	**D**	No	**D**	No	**D**	No	**D**	No
**E**	No	**E**	No	**E**	Yes	**E**	No	**E**	No	**E**	No	**E**	No
**F**	No	**F**	No	**F**	Yes	**F**	Yes	**F**	No	**F**	No	**F**	No
**G**	No	**G**	No	**G**	Yes	**G**	No	**G**	No	**G**	No	**G**	No
**1**	No	**1**	No	**1**	Yes	**1**	Yes	**1**	No	**1**	No	**1**	Yes
**2**	No	**2**	Yes	**2**	Yes	**2**	Yes	**2**	No	**2**	No	**2**	No
**3**	No	**3**	No	**3**	Yes	**3**	No	**3**	No	**3**	No	**3**	Yes

CYP: Cytochrome.
